# Dot1-Dependent Histone H3K79 Methylation Promotes the Formation of Meiotic Double-Strand Breaks in the Absence of Histone H3K4 Methylation in Budding Yeast

**DOI:** 10.1371/journal.pone.0096648

**Published:** 2014-05-05

**Authors:** Mohammad Bani Ismail, Miki Shinohara, Akira Shinohara

**Affiliations:** Institute for Protein Research, Graduate School of Science, Osaka University, Suita, Osaka, Japan; Oklahoma Medical Research Foundation, United States of America

## Abstract

Epigenetic marks such as histone modifications play roles in various chromosome dynamics in mitosis and meiosis. Methylation of histones H3 at positions K4 and K79 is involved in the initiation of recombination and the recombination checkpoint, respectively, during meiosis in the budding yeast. Set1 promotes H3K4 methylation while Dot1 promotes H3K79 methylation. In this study, we carried out detailed analyses of meiosis in mutants of the *SET1* and *DOT1* genes as well as methylation-defective mutants of histone H3. We confirmed the role of Set1-dependent H3K4 methylation in the formation of double-strand breaks (DSBs) in meiosis for the initiation of meiotic recombination, and we showed the involvement of Dot1 (H3K79 methylation) in DSB formation in the absence of Set1-dependent H3K4 methylation. In addition, we showed that the histone H3K4 methylation-defective mutants are defective in SC elongation, although they seem to have moderate reduction of DSBs. This suggests that high levels of DSBs mediated by histone H3K4 methylation promote SC elongation.

## Introduction

Germ cells undergo meiosis to generate haploid gametes. Meiosis involves two consecutive chromosome segregations following one round of DNA replication. During meiosis I, homologous chromosomes segregate to opposite poles, and during meiosis II, as in mitosis, sister chromatids are separated [1]. Physical linkages between the homologous chromosomes ensure the proper segregation of the chromosomes during meiosis I. This physical linkage is cytologically visualized as the chiasma. The formation of chiasmata requires exchanges between parental homologous chromosomes, products of homologous recombination during meiosis [2].

Meiotic recombination occurs at distinct regions of the genome, called recombination hotspots [3], [4]. The hotspots are distributed non-randomly along chromosomes. The recombination is initiated by the formation of double-strand breaks (DSBs) at the hotspot by a meiosis-specific topoisomerase II-like protein, Spo11, and its associated partner proteins [3], [4]. Meiotic DSB formation in yeast often occurs in intergenic regions, which are depleted in nucleosomes [5], [6]. Meiotic recombination hotspots are marked with histone post-translational modifications such as histone H3K4 methylation in budding yeast and mammals, and histone H3K9 acetylation in fission yeast [7]–[9]. Histone H3K4 methylation at the hotspot is catalyzed by Set1 and Prdm9 methyltransferases in budding yeast and in mammals, respectively [8], [10]. Deletion of Set1 in the yeast reduces DSB formation and changes its distribution, and Prdm9 knockout in mouse changes the distribution of DSBs across the genome [11], [12]. Indeed, substitution of histone H3K4 modulates DSB formation, as seen in the *set1* mutant [12], [13]. Moreover, Spp1, a component of the Set1 complex (COMPASS), recognizes H3K4 methylation through its PHD finger and binds to a Spo11 partner, Mer2, by tethering the hotspot located in chromatin loops to the chromosome axis-associated DSB machinery [12], [13]. Importantly, the *set1* mutant of yeast and *prdm9* mutant mice still show significant residual DSB formation, and therefore show meiotic recombination [7], [11]. The yeast *set1* mutant affects DSB distribution with creation of new recombination hotspots [7], suggesting the presence of an alternative pathway for DSB formation. How the formation of these residual DSBs is promoted in the absence of H3K4 methylation remains unsolved.

DSBs are processed to generate 3′-OH over-hanged single-stranded DNAs (ssDNA). This ssDNA is engaged in the interaction with intact duplex DNA on a homologous chromosome. Once homology is matched, the ssDNA invades the duplex DNA to form a recombination intermediate; this is called single-stranded invasion (SEI) [14]. The homology search and strand exchange is dependent on two RecA homologs, Rad51 and Dmc1, particularly Dmc1 [15]–[17]. The SEI is then converted into an intermediate with two Holliday junctions, called a double-Holliday junction (dHJ) [18]. The dHJ is then preferentially resolved into a crossover product for a chiasma. Non-crossovers are formed through an early-branched pathway prior to SEI and dHJ formation [14], [19].

During this recombination, a meiotic cell undergoes drastic changes in chromosome structures [20]. One prominent meiosis-specific chromosome structure is the synaptonemal complex (SC), which has a zipper-like morphology. Two sister chromatids are tightly connected to form a chromosome axis. In the SC, 2 chromosome axes from homologous chromosomes pair with each other through transverse filaments between the axes. Chromosome axis structures in SCs are referred to as axial/lateral elements. The formation of SCs is tightly coupled with ongoing recombination in the budding yeast [21].

It has been proposed that meiotic recombination and possibly SC formation are subject to surveillance. One of the surveillance mechanisms is a coupling mechanism of the meiotic events with cell cycle progression, which is often referred as to the pachytene checkpoint or the recombination checkpoint [22]. This surveillance mechanism has been studied extensively using mutants defective in meiotic recombination and/or SC formation; e.g., the *dmc1* mutant for meiotic recombination and *zip1* mutant for SC formation [15], [23]. These mutants show delay or arrest in entry into meiosis I. When recombination is defective, meiotic cells cannot exit the middle of the pachytene phase. This is due to an inability of the mutant cell to express the Ndt80 transcriptional activator [24], which promotes the expression of so-called “middle sporulation” genes such as Cdc5 polo-like kinase and Clb1 cyclin for exit from the pachytene phase [25]. Increased Cdc5 as well as increased Cdk1 activities are key to exiting the mid-pachytene phase for SC disassembly and resolution of dHJs [26], [27]. Genetic screens have identified several mutations that suppress meiotic cell progression delay/arrest by the *dmc1* or *zip1* mutations. Mutations of the *DOT1*(*PCH1*) and *PCH2* genes have been found to alleviate arrest in the *zip1* mutant [28], [29]. The *PCH2* gene encoding a meiosis-specific AAA^+^ ATPase is also involved in chromosome morphogenesis and recombination [28], [30], [31]. The *DOT1* gene encodes a histone H3K79 methyltransferase which is required for gene silencing and control of some DNA damage repair pathways in mitosis [32]–[36]. Interestingly, both Set1-dependent H3K4 methylation and Dot1-dependent H3K79 methylation are promoted by the Rad6/Bre1-dependent ubiquitination of the histone H2BK123 [37], [38]. In meiosis, H2BK123 ubiquitination is also important for DSB formation and for timely entry into meiosis I [39].

In this study, we analyzed the role of Set1 and Dot1 histone H3 methyltransferases in DSB formation and SC formation during meiosis. Consistent with previous studies, the *set1* mutant reduces DSBs on the genome as revealed by immunostaining studies for Rad51 foci. Surprisingly, *set1* deletion or H3K4 methylation-defective mutants still retain two-thirds the levels of Rad51 foci, and thus presumably DSBs, compared to those in the wild type. This suggests the presence of additional determinants in hotspots for DSB formation. Indeed, we find that Dot1-dependent H3K79 methylation is critical for the efficient formation of DSBs in the absence of Set1. Therefore, there might be multiple histone modifications controlling the formation of meiotic DSBs. These studies reinforce the importance of histone posttranslational modifications for chromosome dynamics during meiosis.

## Materials and Methods

### Strains and plasmids

All strains described here are derivatives of the *S. cerevisiae* SK1 diploid strain NKY1551 (*MATα/MAT* ***a*** *, HO::LYS2/”, lys2/”, ura3/”, leu2::hisG/”, his4X-LEU2-URA3/his4B-LEU2, arg4-nsp/arg4-bgl*). The genotypes of each strain used in this study are described in Table S1. The *hht1-K4R hht2-K4R* mutant was constructed by PCR-based mutagenesis. Briefly, wild-type *HHT1* and *HHT2* genes were cloned onto pBluescript II KS+ (Stratagene). PCR-based site-directed mutagenesis using mutant primers was carried out and the presence of the mutation was confirmed by DNA sequencing. The *hht1-K4R hht2-K4R* mutant genes were cloned into YIp*lac*211 and pRS406, respectively. After digestion with *Kpn*I, the DNA was integrated by transformation. The *URA3* gene was popped-out by counter-selection for the *ura* ^−^ phenotype on a 5-FOA plate. Mutant sequences were verified by DNA sequencing using genomic DNAs for candidates. The *hht1-K79R hht2-K79R* strain was a generous gift from Dr. Takehiko Usui. The primers for strain construction are shown in Table S2.

### Cytological analysis and antibodies

Immunostaining was conducted as described [40]. Stained samples were observed using an epifluorescence microscope (BL51; Olympus, Tokyo, Japan) with a 100× objective (NA1.4). Images were captured by CCD camera (Cool Snap; Photometrics) at room temperature, and then processed using iVision (Sillicon, California) software. Pseudo-coloring was performed using Photoshop (Adobe) software. At each timepoint, about 100 spreads were analyzed for counting foci. Primary antibodies directed against Rad51 (guinea pig, 1∶500 dilution), Dmc1 (rabbit, 1∶500 dilution), Zip1 (rabbit, 1∶1000 dilution), Red1 (chicken, 1∶400 dilution), and Rec8 (rabbit, 1∶1000 dilution) were used. Secondary antibodies (Alexa-fluor-488 and -594, Molecular Probes, Carlsbad, CA) directed against primary antibodies from the different species were used at a 1∶2000 dilution. Open-reading frames of Hop1 were PCR-amplified and inserted into a pET21a plasmid (Novagen) in which the C-terminus was tagged with hexahistidine (His6). Fusion proteins with His6 were affinity-purified on nickel/cobalt columns, which was performed by the manufacturers, and used for immunization of guinea pig (MBL Co. Ltd, Nagoya, Japan). The resulting antibody preparation was used at a 1∶1000 dilution for western blotting and at a 1∶500 dilution for immunostaining. A monoclonal antibody directed against the ***α***-subunit of rat tubulin was also used (AbD Serotec, Oxford, UK). Meiotic time course analysis for cytology was carried out 3 times and a representative result is shown.

### Southern and western blotting

For western blotting, cell precipitates were washed twice with 20% (w/v) trichloroacetic acid (TCA) and then disrupted using a bead beater (Yasui Kikai Co. Ltd., Osaka, Japan). Precipitated proteins were recovered by centrifugation and then suspended in sodium dodecyl sulfate polyacrylamide gel electrophoresis (SDS-PAGE) sample buffer. After adjusting the pH to 8.8, samples were incubated at 95°C for 2 min. Antibodies against Cdc5 (sc-33625, SantaCruz), Clb1 (sc-50440, SantaCruz), Hop1, Zip1, Rec8, Red1, and the ***α***-subunit of rat tubulin (Serotec, UK) were used. Antibodies against histone H3K4-me3 (ab8580) and H3K79-me3 (ab2621) were from Abcam (Cambridge, UK).

For Pulse Field Gel Electrophoresis (PFGE), DNAs were prepared in agarose plugs as described [41], and run under the condition (120°, 14C°, 46 h at 6 V/cm) by CHEF DR-III (BioRad). Switching time was 25 to 125 seconds.

Southern blotting was performed as described previously [42], [43]. For the *HIS4-LEU2* locus, genomic DNA was digested using *Mlu*I and *Xho*I (for CO and NCO) or *Pst*I (for meiotic DSB). For the *YCR048W* locus, the DNA was digested with *Bgl*II. Probes for Southern blotting were “Probe 155” for CO/NCO, and “Probe 291” for DSB detection at the *HIS4-LEU2* locus [43]. For DSBs at the *YCR047C/048W* locus, a probe for the *YCR052W* locus (215426-216686) was used. For DSBs along chromosome III and VII, *CHA1* and *CUP2* were used as a probe, respectively. Image Gauge software (Fujifilm Co. Ltd., Tokyo, Japan) was used to quantify bands.

## Results

### Set1 and Dot1 play differential roles during meiosis

**Figure 1:**
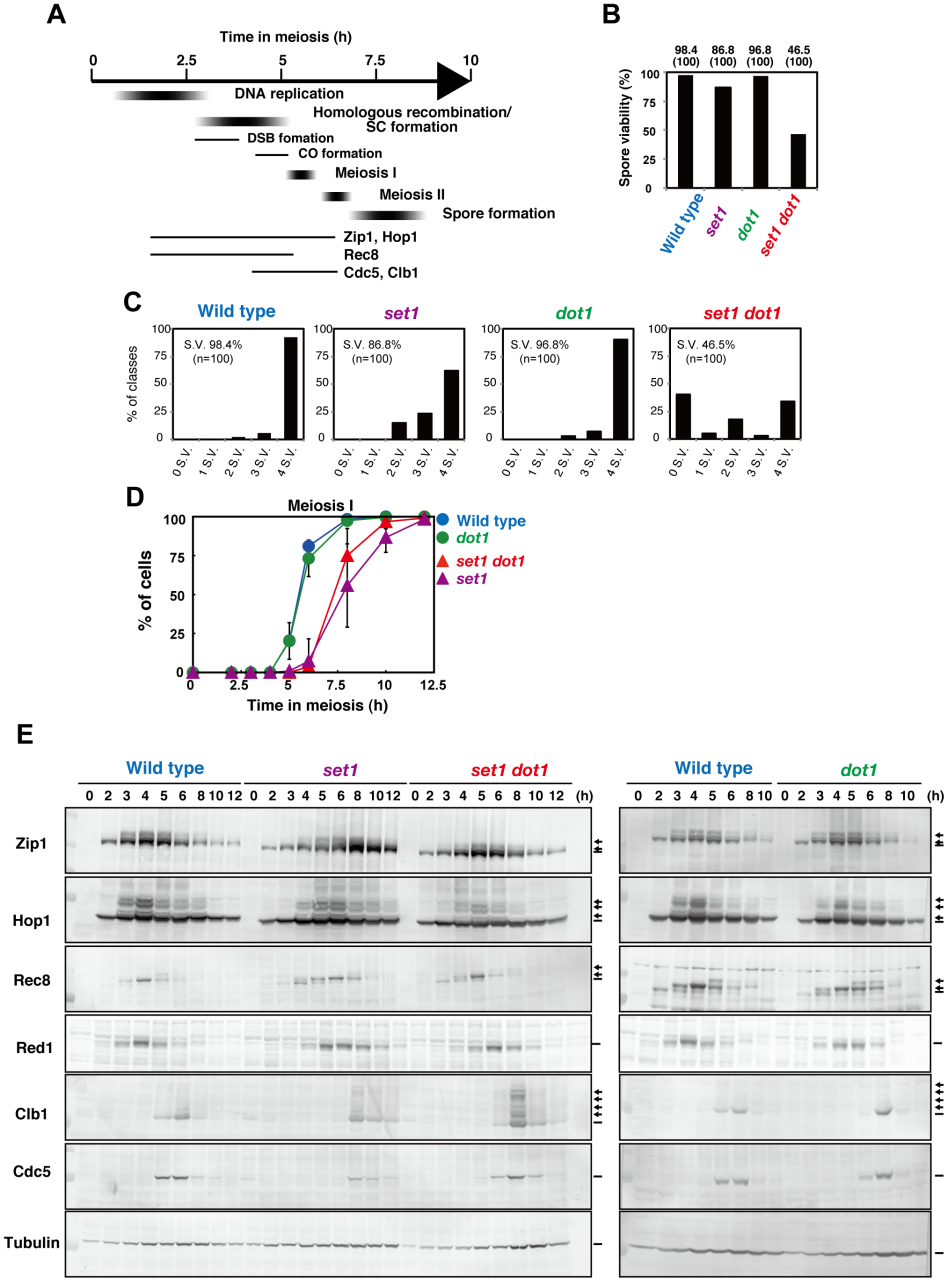
Dot1 plays a meiotic role in the absence of Set1. (A) Schematic representation of events during meiosis. (B, C) Spore viability of various strains was measured by dissecting spores. Spores were incubated at 30°C for 3 days. Each bar indicates percentage of spore viability and actual number of total dissected tetrads (parenthesis). Distribution of viable spores per tetrad is shown in (C). Wild type, NKY1303/1543; *set1* mutant, MBY015/016; *dot1* mutant, MBY005/006; *set1 dot1* double mutant, MBY037/039. (D) Meiotic cell division I was analyzed by DAPI staining of wild type (blue circles; NKY1303/1543), *dot1* (green circles; MBY005/006), *set1* (purple triangles; MBY005/006 and *set1 dot1* (red triangle; MBY037/039) mutant cells. At least 150 cells were counted by DAPI staining for each time point. Plotted values are the mean values with standard deviation (S.D.) from four independent time courses. (E) Expression of various meiotic proteins was verified by western blotting. At each time point, cells were fixed with TCA and cell lysates were subject to the analysis. Representative images are shown. Phosphorylated species of Zip1, Hop1, Rec8, and Clb1 are shown by arrows. Wild type, NKY1303/1543; *set1*, MBY015/016; *dot1*, MBY005/006; *set1 dot1* double mutant, MBY037/039.

Previous studies established the role of Set1-mediated histone H3K4 methylation in DSB formation and the role of Dot1-mediated histone H3K79 methylation in signaling for defective SC formation [10], [29]. To understand the role of these methyltransferases in events during meiosis, we characterized the meiotic phenotypes of the *set1* and *dot1* single mutants, and the *set1 dot1* double mutant in the SK1 background, which confers synchronous meiosis (Figure 1A). As shown previously [29], the *dot1* single mutant exhibits wild-type spore viability. On the other hand, the *set1* single mutant shows a slight reduction to 86.8%, compared to 98.4% in the wild type (Figure 1B). This is different from a published result in which spore viability in the *set1* deletion mutant is not different from that in wild type [12]. The *set1 dot1* double mutant shows a synergistic decrease in viability to 46.5% compared to either single mutant, indicating that Set1 and Dot1 work independently in meiosis. Importantly, the distribution of viable spores per tetrad indicated that the double mutant is more biased towards 4-, 2-, and 0-viable spores rather than 3- and 1-viable spores (Figure 1C), suggesting non-disjunction of homologous chromosomes at meiosis I, which is caused by a defect in meiotic prophase-I. However, among 122 2-spore-viable tetrads, we found that only 1 spore was non-mater, which is indicative for non-disjunction of chromosome III, indicating that non-disjunction of chromosome III is not elevated in the mutant (see Discussion).

4′,6-Diamidino-2-phenylindole (DAPI) staining reveals that the *dot1* mutant shows wild-type like entry into meiosis I (Figure 1D). As reported [10], [12], the *set1* single mutant delays the entry of meiosis I by 2 h compared to wild type, which is mainly caused by delay in the meiotic S-phase [10]. The *set1 dot1* double mutant cells exhibit similar delay to the *set1* single mutant, although the double mutant is more heterogeneous in synchronous progression of the meiotic division than the *set1* single mutant.

We also studied the expression of various proteins in the meiotic prophase, including the SC components Zip1, Hop1, Red1, and Rec8, as well as the pachytene marker proteins Clb1 cyclin and Cdc5 polo-like kinase (Figure 1E). Consistent with the DAPI analysis described above, western blot analysis showed that, in wild type, the appearance of Clb1 and Cdc5 is consistent with decrease of Rec8 level, which is roughly consistent with the entry into MI. The *dot1* mutant shows similar expression pattern of Hop1, Red1, Cdc5 and Clb1 to wild type with slight delayed disappearance of Rec8. The *set1* mutant shows normal appearance of Hop1 and Zip1, but a ∼1-h delay in the appearance of phosphorylated Hop1 and phosphorylated Zip1, a ∼3-h delay in the appearance of Cdc5 and Clb1, a ∼3-h delay in the disappearance of Rec8 and more than 3-h delay in the disappearance of Zip1, compared to the wild type. Like the *set1* mutant, the *set1 dot1* double mutant shows normal appearance of Hop1 and Zip1, but a ∼1-h delay in the appearance of phosphorylated Hop1 and phosphorylated Zip1. Importantly, the double mutant exhibits ∼1-h delay in appearance of Cdc5 and Clb1 compared to wild type, but about 2 h earlier appearance than the *set1* mutant. Consistent with this, disappearance of Zip1, Rec8, and phospho-Hop1 in the double mutant is earlier than the *set1* single mutant. These could be explained by the role of Dot1 in coupling of recombination with exist of pachytene in the absence of Set1 (see below). This is consistent with the role of Dot1 in the pachytene checkpoint when the recombination is perturbed [28], [29].

**Figure 2:**
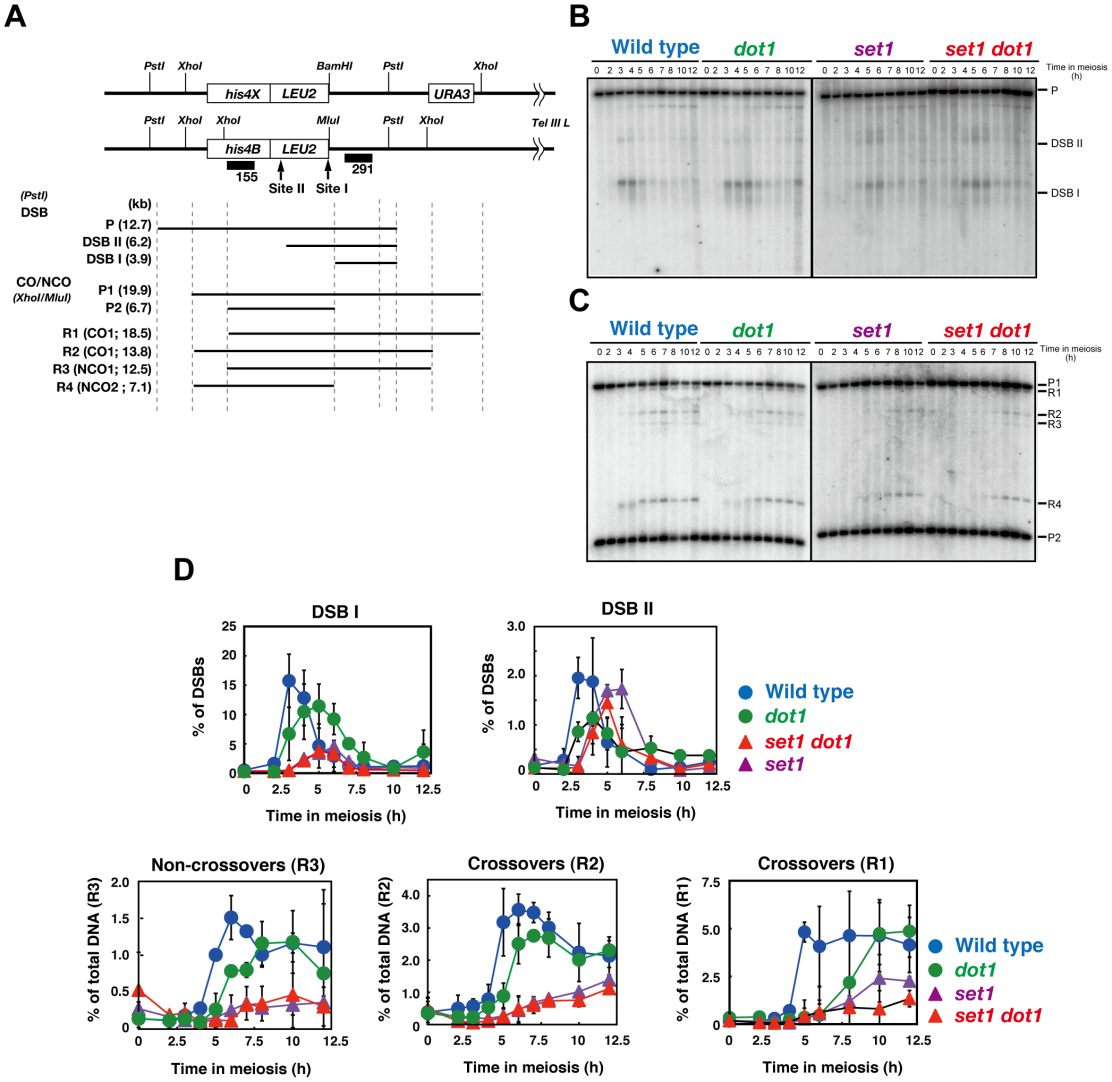
Set1 is necessary for meiotic recombination. (A) Schematic representation of the *HIS4-LEU2* locus. Sizes of fragments for DSB and recombinant analysis are shown with lines below. (B) DSB formation and repair at the *HIS4-LEU2* locus in different strains were verified by Southern blotting. The experiments were independently performed several times and representative blots are shown. Genomic DNAs were digested with *Pst*I. (C) Formation of crossovers and no-crossovers was also analyzed. The experiments were independently performed several times and representative blots are shown. Genomic DNAs were digested with *Mlu*I and *Xho*I. (D) The bands of DSB I (top left) and DSB II (top right), R1 (crossovers; CO; bottom right), R2 (CO; bottom middle) and R3 (non-crossovers; NCO; bottom left) and were quantified. The symbols represent the wild type (blue circles; NKY1303/1543), *dot1* mutant (green circles; MBY005/006), *set1* mutant (purple triangles; MBY015/016) and *set1 dot1* mutant (red triangle; MBY037/039). Plotted values are the mean values with standard deviation (S.D.) from three independent time courses.

In order to know the role of Set1 and Dot1 in meiotic recombination, we studied DSB repair and recombinant formation at a recombination hotspot, the *HIS4-LEU2* locus (Figure 2A) [44]. In the wild type, DSB starts at 2 h, peaks at 3 h, and then gradually disappears (Figure 2B and 2D). The *dot1* mutant exhibits slight delay in the formation of DSBs and delay in the DSB repair relative to the wild type. As reported [10], the *set1* mutant shows a delay in DSB appearance by ∼2 h and a peak at 5 h with reduced steady-state levels of DSBs at site I to 18% of the levels seen in the wild type (at 3 h vs. 5 h in the *set1*). This confirms the role of Set1 in efficient DSB formation [7], [10]. The *set1 dot1* double mutant exhibits similar kinetics to those seen in the *set1* single mutant. The double mutant shows a similar level of steady-state DSBs as seen in the *set1* single mutant, suggesting that Dot1 does not play a role in DSB formation at the *HIS4-LEU2* locus in the absence of Set1.

Using restriction site polymorphisms present on 2 parental DNAs, formation of both CO (R1 and R2) and NCO (R3) was assessed at the *HIS4-LEU2* locus [43] (Figure 2A). The *dot1* mutant exhibits delayed formation of both COs and NCOs by 1–2 h relative to wild type, but the CO and NCO levels in the mutant are almost similar to those in the wild type (Figure 2C and 2D). The *set1* single mutant shows a delay in the formation of recombinants by 3 h and decreases COs (R2) to ∼35% and NCOs (R3) to ∼25% of the levels in the wild type (at 7 h), supporting a role for Set1 in efficient meiotic recombinant formation. The levels of the 2 recombinants in the *set1 dot1* double mutant are almost indistinguishable from those in the *set1* single mutant (Figure 2D).

### Dot1 plays a role in DSB formation in the absence of Set1

**Figure 3:**
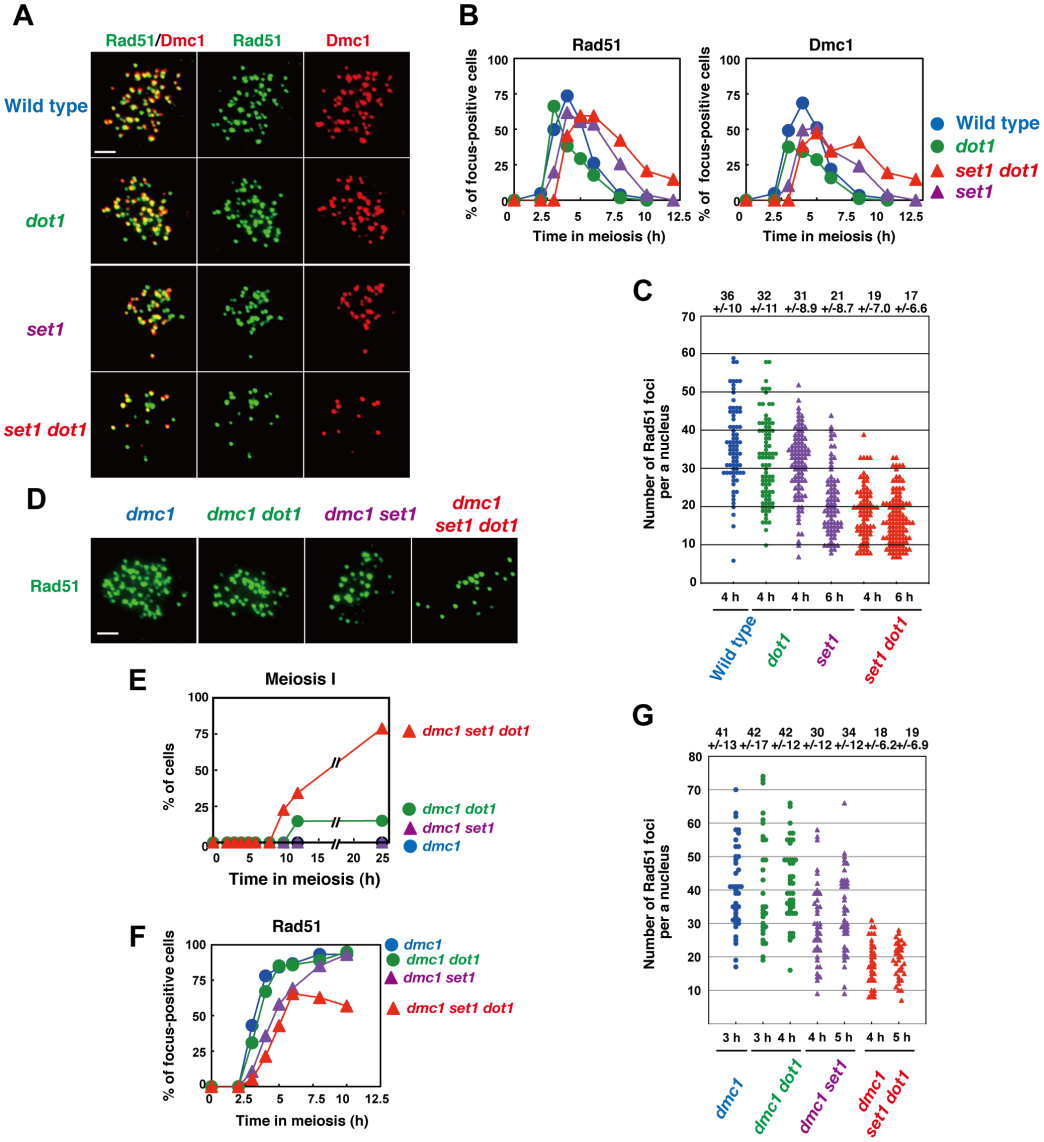
Dot1 promotes Rad51-focus formation in the absence of Set1. (A) Immunostaining analysis of Rad51 (green) and Dmc1 (red) for wild type (NKY1303/1543), *dot1* (MBY005/006), *set1* (MBY015/016) and *set1 dot1* (MBY037/039) mutant strains was carried out. The bar indicates 2 µm. (B) Kinetics of Rad51 (left) or Dmc1 (right)-focus positive cells in various strains. A focus-positive cell was defined as a cell with more than 5 foci. More than 100 nuclei were counted at each time point. The symbols represent the wild type (blue circles; NKY1303/1543), *dot1* mutant (green circles; MBY005/006), *set1* mutant (purple triangles; MBY015/016), and *set1 dot1* mutant (red triangle; MBY037/039). (C) A number of foci of Rad51 were counted in different strains. The symbols represent the wild type (blue circles; NKY1303/1543), *dot1* mutant (green circles; MBY005/006), *set1* mutant (purple triangles; MBY015/016), and *set1 dot1* mutant (red triangle; MBY037/039). The average number of foci per positive nucleus with S.D. is shown on top. (D) Immunostaining analysis of Rad51 (green) for the *dmc1* mutant (MBY009/010), *dmc1 dot1* mutant (MBY003/004), *dmc1 set1* mutant MBY021/022), and *dmc1 set1 dot1* mutant (MBY282/285) was carried out. The bar indicates 2 µm. (E) Meiotic cell division I was analyzed by DAPI staining of the *dmc1* mutant (blue circles; MBY009/010), *dmc1 dot1* mutant (green circles; MBY003/004), *dmc1 set1* mutant (purple triangles; MBY021/022), and *dmc1 set1 dot1* mutant (red triangle; MBY282/285) cells. At least 150 cells were counted by DAPI staining for each time point. (F) Kinetics of Rad51-focus positive cells in the *dmc1* mutant (blue circles; MBY009/010), *dmc1 dot1* mutant (green circles; MBY003/004), *dmc1 set1* mutant (purple triangles; MBY021/022), and *dmc1 set1 dot1* mutant (red triangle; MBY282/285) strains. A focus-positive cell was defined as a cell with more than 5 foci. More than 100 nuclei were counted at each time point. (G) The number of Rad51 foci was counted in different strains as described above. The average numbers of foci per a Rad51-foci positive nucleus with S.D. is shown on top.

To address the role of Set1 and Dot1 in the formation and repair of DSBs across the genome, we carried out immunostaining analysis for Rad51, a RecA homolog [40], involved in both mitotic and meiotic recombination, and the meiosis-specific RecA homolog, Dmc1 [15]. The collaboration of Rad51 and Dmc1 is key to interhomolog recombination [16], [45], [46]. As shown previously [47], Rad51 shows punctate staining on meiotic chromosomes (Figure 3A). Rad51 foci correspond with sites of ongoing recombination [40], [48]. Counting of nuclei positive for Rad51 foci (more than 5 foci) shows the kinetics of DSB repair (Figure 3B). The *dot1* mutant shows the similar kinetics of Rad51-focus appearance as seen in the wild type. However, the disappearance of Rad51 foci occurs earlier in the *dot1* mutant than the wild type. The appearance of Rad51 foci in the *set1* mutant is delayed by about 2 h relative to the wild type, consistent with the delay of the onset of the pre-meiotic S phase in the mutant. On the other hand, disappearance of Rad51 foci in the mutant shows a ∼3-h delay compared to the wild type. If the S-phase delay is accounted for [10], the *set1* mutant delays Rad51-focus disassembly by about 1 h, suggesting a role for Set1 in DSB repair in meiosis. The *set1 dot1* double mutant shows delayed appearance of Rad51 similar to the *set1* single mutant. However, the disappearance of Rad51 foci in the double mutant is 1 h later than that in the single mutant. This suggests a role for Dot1 in meiotic DSB repair in the absence of Set1.

Numbers of Rad51 foci per focus-positive cell are indicative of the steady-state numbers of DSBs in a cell (Figure 3C). The average numbers of foci in the wild type and the *dot1* mutant at 4 h are 36±10 (n  =  78) and 32±12 (n  =  89), respectively (P value  =  0.01; Mann-Whitney's *U*-test). The *set1* mutant shows a slightly reduced number of foci (31±8.9; n  =  95; 86% of wild-type level) at 4 h and more reduced number (21±8.8; n  =  79; 58% of wild-type level at 4 h) at 6 h, consistent with the reduction of DSBs in this mutant (wild type; P value  = 0.0041, 2.2 × 10^−15^, respectively, Mann-Whitney's *U*-test). Reduced focus number at 6 h compared to that at 4 h may be due to disassembly of Rad51 from chromosomes by DSB repair in the mutant. Moreover, the *set1 dot1* double mutant shows a reduced Rad51-focus number (17±6.6; n  =  116) at 6 h, which is much lower than that in the *set1* single mutant (at 6 h; P value  =  5.3 × 10^−5^), as well as at 4 h (19±7.0; n  =  78; versus at 4 h in the *set1*, P value  =  2.2 × 10^−16^), suggesting a significant role for Dot1 in DSB formation in the absence of Set1. Mutations in the *SET1* and/or *DOT1* genes show similar effects in the kinetic analysis of Dmc1 as those seen with Rad51 foci (Figure 3B).

Given the critical role of Set1-dependent histone H3K4 tri-methylation in DSB formation across genome [7], [12], [13], relatively high numbers of Rad51 foci, thus DSBs, in the *set1* single mutant are a bit surprising. Consistent with significant DSB formation in the *set1* mutant, we observed that steady levels of Hop1 phosphorylation, which depends on DSBs through the activation of Mec1/Tel1 kinases [49], in the *set1* mutant were comparable to those in wild type (shifted bands [arrows] in Figure 1E). To confirm the results, we also counted the number of Rad51 foci in the background of the *dmc1* mutant (Figure 3D), which is defective in the repair of DSBs and, as a result, accumulates the foci [15]. As reported [29], the *dot1* mutation weakly suppresses *dmc1*-induced cell cycle arrest (Figure 3E). Interestingly, the combination of *set1* and *dot1* mutations alleviates *dmc1* arrest to a greater extent than does the *dot1* mutation alone. As expected, all 4 strains with the *dmc1* mutation accumulate Rad51-focus positive cells (Figure 3D and 3F). As with Rad51-focus counting, we analyzed early time points up to 5 h when Rad51 is in the assembly stage. In the *dmc1* and *dot1 dmc1* mutants, the average numbers of Rad51 foci at 3 h are 41±13 (n  =  41) and 42 ±17 (n  =  30), respectively (Figure 3G). The *set1 dmc1* mutant shows a reduced number of foci (34±12; n  =  42) at 5 h (P value  =  1.5 × 10^−4^, versus at 3h in *dmc1*, Mann-Whitney's *U*-test), confirming the role of Set1 in DSB formation, although the effect of the *set1* mutant is 30% reduction compared to wild type. Again, the *set1 dot1 dmc1* triple mutant shows a decreased number of foci, to 19±6.9 (n  =  52) at 5 h, which is much lower than that in the *set1 dmc1* mutant (P value  =  5.6 × 10^−6^, versus *dmc1 set1* at 5 h, Mann-Whitney's *U*-test). This supports a role for Dot1 in DSB formation without Set1.

### Dot1 plays a role in DSB formation at some regions of chromosomes

**Figure 4:**
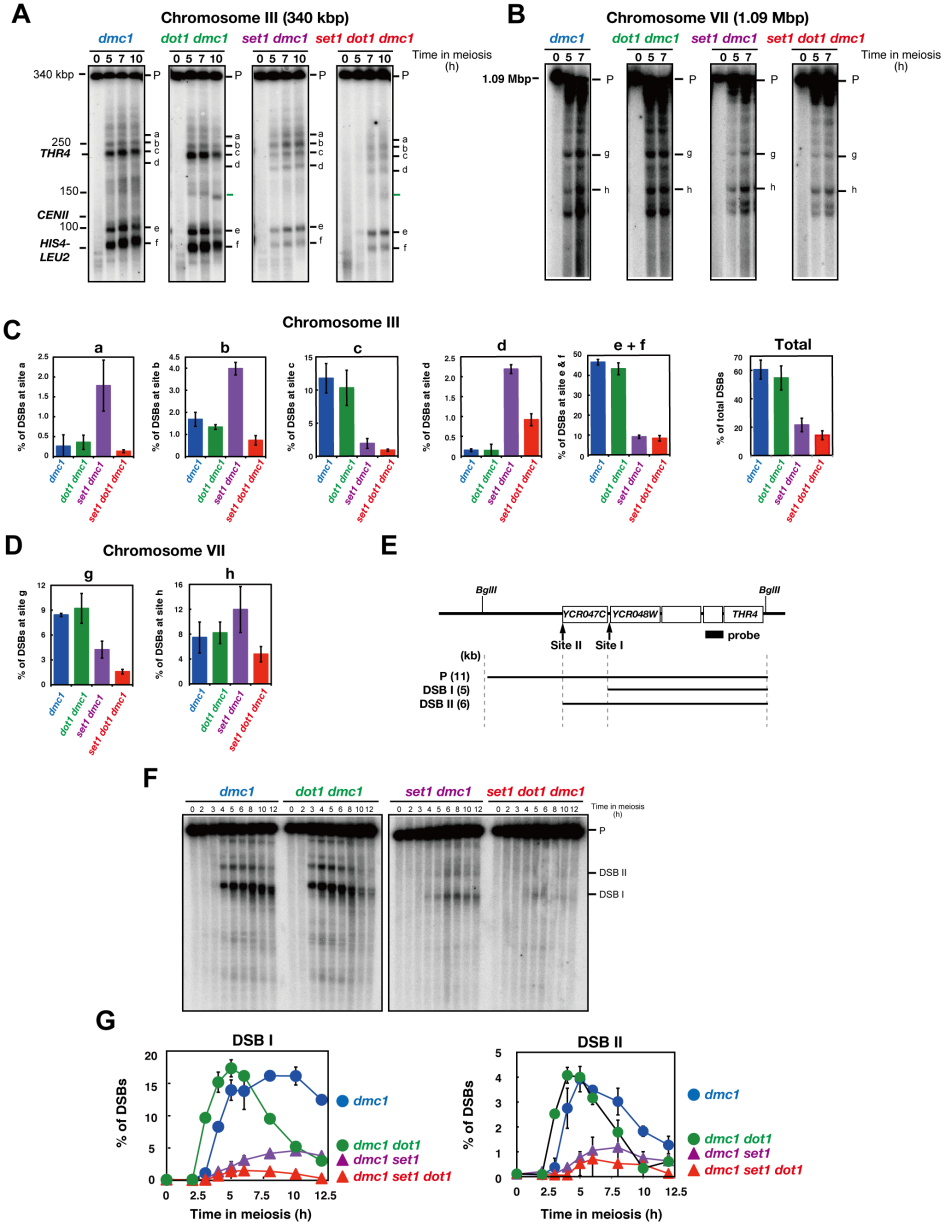
Dot1 promotes the formation of DSBs in the absence of Set1. (A) Distribution of DSBs along Chromosome III was analyzed by PFGE followed by indirect labeling of one chromosome end using the *CHA1*. Samples from meiotic time courses of the *dmc1* mutant (MSY2630/2632), *dmc1 dot1* mutant (MBY003/004), *dmc1 set1* mutant (MBY021/022), and *dmc1 set1 dot1* mutant (MBY282/285) were analyzed. Band positions used for the quantification in (C) are shown on right. On left, approximate size of chromosomes and the position of two recombination hotspots, *HIS4-LEU2* and *THR4* are indicated. Green bars on the right side are a possible “Dot1”-dependent DSB bands. (B) Distribution of DSBs along Chromosome VII was analyzed by PFGE followed by indirect labeling of one chromosome end using the *CUP2*. Band positions used for the quantification in (D) are shown on right. (C) Quantification of DSB frequencies at defined positions on chromosome III were carried out for the *dmc1* mutant (Blue bars, MSY2630/2632), *dmc1 dot1* mutant (Green Bars, MBY003/004), *dmc1 set1* mutant (purple bars, MBY021/022), and *dmc1 set1 dot1* mutant (red bars, MBY282/285). Total amounts of DSBs along the chromosome are also shown in right. Plotted values are the mean values with standard deviation (S.D.) at 7 h from three independent time courses. (D) Quantification of DSB frequencies at defined positions on chromosome VII were carried out as shown in (C). Plotted values are the mean values standard deviation (S.D.) at 7 h from two independent time courses. (E) Schematic representation of the *YCR047C/CR048W* locus. Sizes of fragments for DSB are shown with lines below. (F) DSB formation at the *YCR047C/CR048W* locus in different strains was verified by Southern blotting. Genomic DNAs were digested with *Bgl*II. (G) The bands of DSBs I (left) and II (right) at the *YCR047C/CR048W* locus were quantified. The experiments were independently performed three times and representative blots are shown. The symbols represent the *dmc1* (blue circles; MSY2630/2632), *dmc1 dot1* mutant (green circles; MBY003/004), *dmc1 set1* mutant (purple triangles; MBY021/022) and *dmc1 set1 dot1* mutant (red triangle; MBY282/285). Plotted values are the mean values with standard deviation (S.D.) from three independent time courses.

The above cytological analysis of Rad51 foci across the genome indicates a role of Dot1 for DSB formation in the absence of Set1. On the other hand, physical analysis at the artificial recombination hotspot, *HIS4-LEU2*, did not support this idea (Figure 2D). To know the role of Dot1 in genome-wide DSB formation, we studied the distribution of DSBs on single chromosomes by using pulse-field gel electrophoresis (PFGE) [41]. We analyzed DSB distribution on chromosome III as a representative of small chromosomes of yeast and VII as a long chromosome in the *dmc1* mutant background (Figure 4A and 4B). For the mapping, we used the *dmc1* rather than the *rad50S*, in which Tel1/ATM kinase is activated to down-regulate DSB formation, particularly on long chromosomes [50]. The DSB mapping showed region-specific enrichment of DSBs on chromosome III and VII in the *dmc1* mutant. We also chose several regions hot for DSB formation and quantified the amounts of DSBs at the regions (Figure 4C and 4D). The *dot1* mutant (with the *dmc1*) shows similar patterns of DSB distributions on both chromosomes III and VII with similar DSB formation efficiencies to the control wild type. The *set1* mutant greatly reduces DSB formation along chromosomes with variation in its effect. This is consistent with previous observations by ChIP(Chromatin Immunoprecipitation)-chip [7], [12]. The *set1* mutation not only reduces DSB formation but also increases DSB formation at several loci [7], [12]. Increased levels of DSBs in the *set1 dmc1* mutant are seen at regions, a, b and d on chromosome III compared to the *dmc1* mutant (Figure 4C). Although the *dot1* mutation did not affect DSB formation at regions c and e + f of chromosome III in the absence of the *SET1*, the mutation reduced DSB formation at regions a, b, and d on chromosome III, and g and h on chromosome VII. The positive role of Dot1 in DSB formation is clearly seen in regions in which DSB formation is increased in the absence of Set1. Interestingly, the *dot1* (and *set1 dot1*) mutant shows novel DSB hotspots at a late time point (green bars around ∼150 kb region of chromosome III, right green bar in Figure 4A), supporting a possible role of Dot1 in DSB formation even in normal meiosis.

We also analyzed the role of Dot1 on DSB formation at a single locus. We focused on a hotspot, *YCR047C/YCR048W* locus on chromosome III (Figure 4E). The *dmc1* mutant accumulates DSBs at the *YCR047C/YCR048W* locus (Figure 4F and 4G). On the other hand, DSBs in the *dot1 dmc1* double mutant accumulate as in the *dmc1* mutant but gradually reduce during further incubation. This might be due to either more resection of DSB ends or DNA repair [15]. The *set1 dmc1* double mutant shows decreased DSB levels (for DSB I) to 23% of wild type (at 6 h). Importantly, the *set1 dot1 dmc1* triple mutant reduced the DSB levels to 48% (for DSB I) compared to the *set1 dmc1* double mutant (P = 0.0089, Student's *t*-test). These results support the idea that Dot1 is involved in DSB formation in the absence of Set1.

### Set1 and Dot1 play a role in the formation of synaptonemal complex

**Figure 5:**
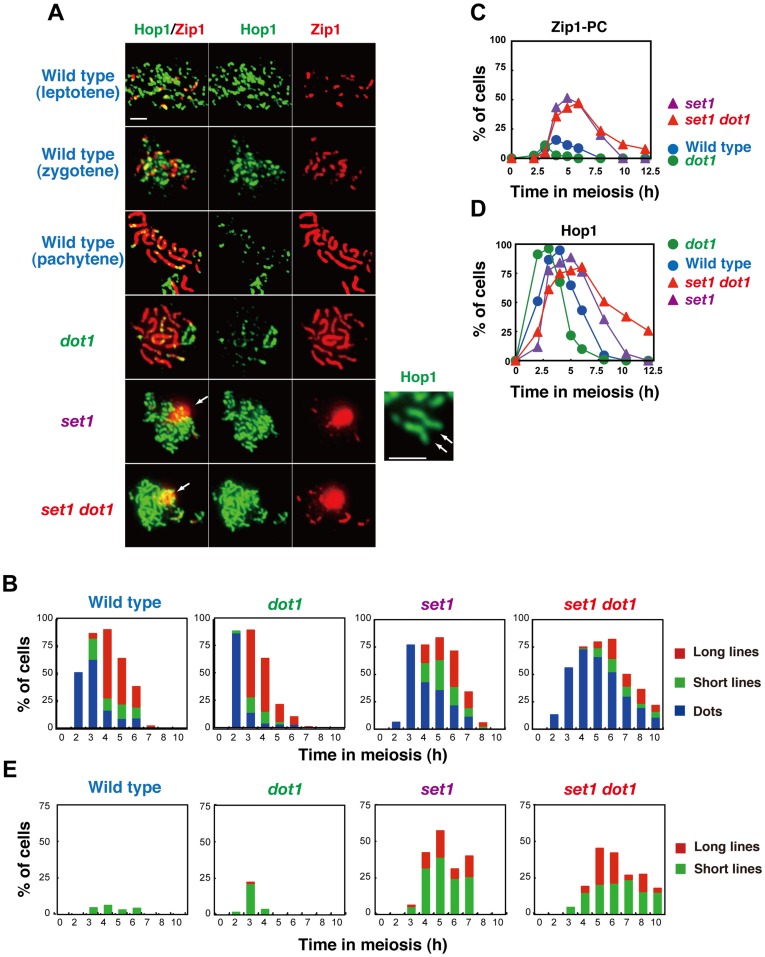
Set1 promotes the formation of synaptonemal complex. (A) Immunostaining analysis of chromosome proteins, Zip1 (red) and Hop1 (green), was carried out for wild type and different mutant strains. Representative images are shown for each strain. Representative images for parallel Hop1 lines in the *set1* mutants are shown in right. White arrows indicate polycomplexes of Zip1. Wild type, NKY1303/1543; *set1* mutant, MBY015/016; *dot1* mutant, MBY005/006; *set1 dot1* double mutant, MBY037/039. The bar indicates 2 µm. (B) Zip1 staining in wild type and mutant strains was classified as follows: dot (dots I, blue), partial linear (short lines, green), full SC (long lines, red). More than 100 nuclei were counted at each time point. Wild type, NKY1303/1543; *set1* mutant, MBY015/016; *dot1* mutant, MBY005/006; *set1 dot1* double mutant, MBY037/039. (C) Kinetics of spreads with Zip1-PCs were analyzed. Wild type, blue circles; *set1* mutant, green circles; *dot1* mutant, purple triangles; *set1 dot1* double mutant, red triangles. (D) Kinetics of spreads positive for Hop1 were verified in different strains. Wild type, blue circles; *set1* mutant, green circles; *dot1* mutant, purple triangles; *set1 dot1* double mutant, red triangles. (E) Hop1-staining in different strains was classified: short lines (green) and long lines (red). Positive cells for each class were counted. More than 100 nuclei were counted at each time point.

Previously, the role of 2 histone H3 methyltransferases in the formation of meiotic chromosome structures had not been described well. We first examined the formation of the synaptonemal complex (SC) by immunolocalization of Zip1, which is a component in the central region of the SC [23]. Zip1 staining was classified into 3 classes: dots, partial lines and full lines, which may roughly correspond with the leptotene, zygotene, and pachytene stages, respectively (Figure 5A). Immunostaining reveals unique contributions of Set1 and Dot1 to SC formation. The *dot1* single mutant shows near wild-type kinetics for Zip1 assembly and disassembly, except that dotty staining of Zip1 appears earlier and Zip1 assembly disappears a bit earlier in the *dot1* cells relative to wild type (Figure 5B).

The *set1* single mutant shows clear defects in SC assembly (Figure 5A and 5B). The appearance of Zip1 dotty staining is delayed by ∼1 h, probably due to a delay in the S-phase. Moreover, the mutant shows reduced frequencies of full-length SCs. Furthermore, Zip1 disassembly occurs ∼1 h later than in wild type, even after compensating for the delay in assembly. Consistent with the defect in Zip1 assembly in the *set1* mutant, the mutant accumulates an aggregate of Zip1, referred to as a polycomplex (PC; Figure 5A and 5C). This confirms previous observation that the *set1* mutant is defective in SC formation [51]. The *set1 dot1* double mutant exhibits more defects in Zip1 elongation (reduced pachytene cells) and a greater delay in Zip1-disassembly than does the *set1* single mutant, suggesting a role for Dot1 in SC formation in the absence of Set1. This SC-defect in the double mutant may be caused by the repair defect and/or reduced DSB formation in the mutant (see above).

To analyze SC defects seen in the *set1* mutant in more detail, we also examined the localization of Hop1 (Figure 5A), which is a component of the chromosome axis and is required for SC formation as well as meiotic recombination [52]. In the wild type, Hop1 appearance occurs as early as 2 h after the induction of meiosis, and Hop1 disappearance takes place around the pachytene stage; e.g., 5 h (Figure 5D). In wild-type cells, Hop1 shows punctate staining in early meiotic prophase I and reduced staining during late prophase (Figure 5A). The *dot1* single mutant shows very similar Hop1 staining patterns to those seen in the wild type, although, as seen for Zip1, Hop1-loading occurs earlier in the mutant than in wild type. Importantly, the *set1* single mutant shows a 1-h delay in the assembly of Hop1 foci relative to the wild type, and a 3-h delay in the disassembly. Moreover, in addition to dotty staining of Hop1, *set1* cells show elongated lines of Hop1, which is rarely seen in the wild type (Figure 5A and 5E). In some nuclei, 2 lines of Hop1 are aligned side-by-side (Figure 5A, shown by a pair of arrows), suggesting that the pairing of homologous chromosomes takes place normally, but full synapsis is impaired in the *set1* mutant. The *dot1 set1* double mutant exhibits very similar patterns with the exception of greater proportions of long Hop1 lines and delayed disappearance of Hop1 from chromosomes relative to the *set1* mutant, consistent with a role for Dot1 in SC formation without Set1.

Consistent with a previous observation [30], double staining of Zip1 and Hop1 clearly shows that Hop1-enriched regions lack strong Zip1 signals in all strains including wild type (Figure 5A), confirming the previous idea that Hop1 in yeast is disassembled along with Zip1 elongation as seen in other eukaryotic organisms, such as Hormad1 in mammals [53] and Asy1 in plants [54]. The accumulated localization of Hop1 along the chromosomes in the *set1* mutant is possibly consistent with the fact that Set1 is required for Zip1 elongation.

**Figure 6:**
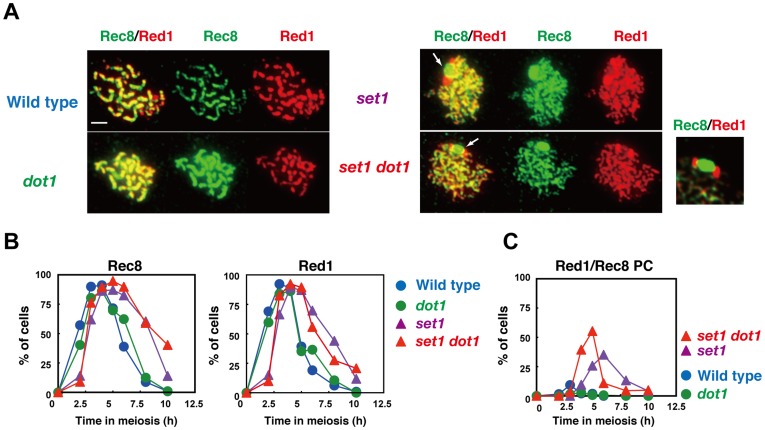
Set1 promotes normal assembly of chromosome axes. (A) Immunostaining analysis of chromosome proteins, Red1 (red) and Rec8 (green), were carried out for wild type and different mutant strains. Representative images for pachytene (wild type *dot1*) and pseudo-pachytene (*set1* and *set1 dot1*) stages are shown for each strain; wild type 5 h; the *dot1*, 5h; the *set1*, 6 h; the *set1 dot1*, 6 h. White arrows in the *set1* or *set1 dot1* mutants shows Rec8/Red1 aggregates. An image for Rec8/Red1 aggregates in the *set1 dot1* mutant is enlarged and shown in right. Wild type, NKY1303/1543; *set1* mutant, MBY015/016; *dot1* mutant, MBY005/006; *set1 dot1* double mutant, MBY037/039. The bar indicates 2 µm. (B) Kinetics of spreads positive for Red1 (right) and Rec8 (left) were verified in different strains. The symbols indicate the wild type (blue circles; NKY1303/1543), *dot1* mutant (green circles; MBY005/006), *set1* mutant (purple triangles; MBY015/016), and *set1 dot1* mutant (red triangle; MBY037/039). (C) Kinetics of spreads with Red1/Rec8-PCs were analyzed. Wild type, blue circles; *set1* mutant, green circles; *dot1* mutant, purple triangles; *set1 dot1* double mutant, red triangles.

We also analyzed the localization of another axis protein, Red1 [55], as well as that of the meiosis-specific kleisin, Rec8, which is a component of the cohesion complex [56](Figure 6). Red1 works together with Hop1 as well as with Mek1/Mre4 in both meiotic recombination and chromosome morphogenesis [57], [58]. Red1 initially appears as focal staining like Hop1, but later, unlike Hop1, it forms discontinuous lines as the SC elongates (Figure 6A and 6B) [59]. In the *set1* and *set1 dot1* mutants, both of which shows delay in the assembly and disassembly of Red1 (Figure 6B), there are little thick Red1 lines, consistent with defective SC elongation in the mutants. Rec8 localization is similar to that of Red1 in wild type and *dot1* strains. On the other hand, the *set1* and *set1 dot1* mutants rarely form thick lines of Rec8 as seen in the wild-type and *dot1* mutant cells, consistent with the lack of full SCs in the mutants (Figure 5A). Notably, we observed aggregates of Red1 and Rec8 in the *set1* and *set1 dot1* mutants (Figure 6A). At 6 h, about 35% of *set1* mutant cells contain Red1 and Rec8 aggregates (Figure 6C). This number is increased to 55% at 5 h in the *set1 dot1* double mutant. In the PC-like structure, Red1 shows bipolar staining on a large Rec8-block (Figure 6A). It is important to point out that these Red1 and Rec8 aggregates are not formed in other SC-deficient mutants such as the *dmc1* mutant. These results show that Set1 is important for SC elongation and that Dot1 plays a role in SC formation only in the absence of Set1. Set1 may be important for the organization of the chromosome axis containing Red1 and Rec8 for synapsis. On the other hand, recent ChIP-chip study shows that the *set1* mutant shows wild-type distribution of Rec8 along chromosomes [12]. Therefore, more higher order structure of chromosome axes might be compromised in the mutant.

### The histone H3K4 mutant is defective in SC formation

**Figure 7:**
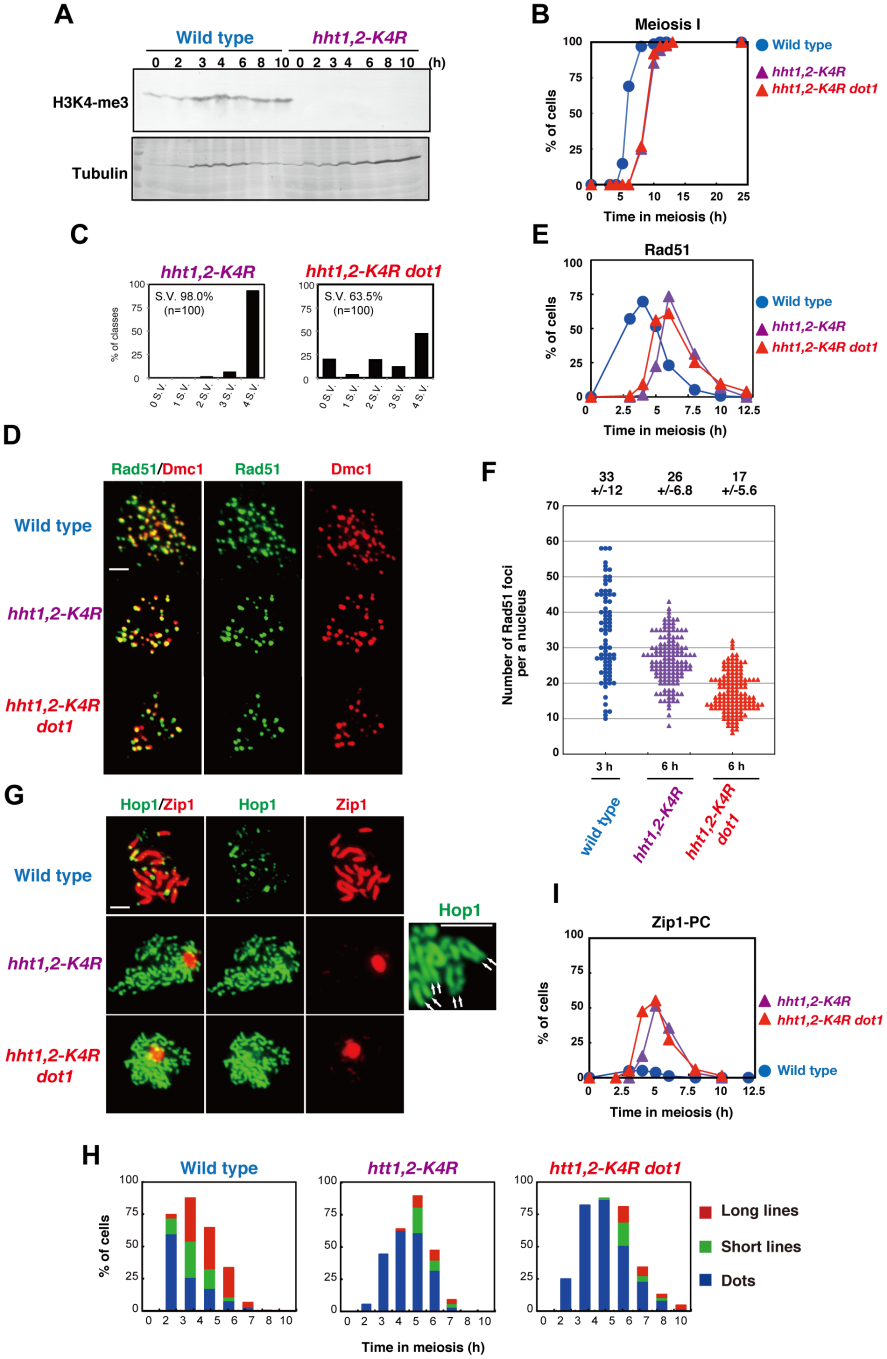
Histone H3K4 is critical for DSB and SC formation. (A) Expression of histone H3K4 trimethylation during meiosis. Western blotting analysis for wild type (NKY1303/1543), *hht1,2-K4R* (MBY211/218) was carried out using anti-histone H3K4-me3. (B) Meiotic cell division I was analyzed by DAPI staining of wild-type (blue circles; NKY1303/1543), *hht1,2-K4R* (purple triangles; MBY211/218), and *hht1,2-K4R dot1* (red triangles; MBY233/237) strains. At least 150 cells were counted by DAPI staining for each time point. (C) Distribution of viable spores per tetrad in wild-type and *hht1,2-K4R dot1* (MBY233/237) strains. For each strain, 100 tetrads were dissected. (D) Immunostaining analysis of Rad51 (green) and Dmc1 (red) for wild type (NKY1303/1543), *hht1,2-K4R* (MBY211/218), and *hht1,2-K4R dot1* (MBY233/237) strains was carried out. The bar indicates 2 µm. (E) Kinetics of Rad51 focus-positive cells in various strains. A focus-positive cell was defined as a cell with more than 5 foci. More than 100 nuclei were counted at each time point. The symbols indicate the wild type (blue circles; NKY1303/1543), *hht1,2-K4R* (purple triangles; MBY211/218), and *hht1,2-K4R dot1* (red triangles; MBY233/237) strains. (F) Rad51 focus numbers per nucleus were counted in different strains. The symbols indicate the wild type (blue circles; NKY1303/1543), *hht1,2-K4R* (purple triangles; MBY211/218), and *hht1,2-K4R dot1* (red triangle; MBY233/237) strains. The average number of foci is shown per positive nucleus. (G) Representative images for staining of Zip1(red) and Hop1(green) in wild-type and mutant strains are shown. Hop1 parallel lines the *hht1,2-K4R* mutant are shown in a pair of arrows on the right. The bar indicates 2 µm. (H) Zip1-staining was classified into 3 classes: dot (dots, blue), partial linear (short lines, green), and full SC (long lines, red). More than 100 nuclei were counted at each time point Kinetics of spreads (Zip1 polycomplexes) were analyzed. Wild type, NKY1303/1543; *hht1,2-K4R*, MBY211/218; *hht1,2-K4R dot1*, MBY233/237. (I) Kinetics of Zip1-PC in different strains. The number of spreads containing Zip1-PC was counted in each strain. The symbols indicate the wild type (blue circles; NKY1303/1543), *hht1,2-K4R* (purple triangles; MBY211/218), and *hht1,2-K4R dot1* (red triangle; MBY233/237) strains.

Set1 is a H3K4 methyltransferase [38]. To confirm the role of Set1 in meiotic chromosome metabolism through this histone modification, we constructed a *hht1-K4R hht2-K4R* double mutant (hereafter, *hht1,2-K4R*) at the native chromosomal loci. This strain construct is different from a previous strain, in which both *HHT1* and *HHT2* were deleted, but an *ARS-CEN* plasmid with the *hht1-K4R hht2-K4R* mutations were present [13]. The absence of H3K4 tri-methylation was confirmed by western blotting (Figure 7A). The *hht1,2-K4R* double mutant shows wild-type spore viability (Figure 7C). This is different from slight reduction of spore viability of the *set1* mutant. The *hht1,2-K4R* cells show a greater delay (∼3 h) in the entry into meiosis I than does the *set1* single mutant with ∼2 h delay (Figure 7B). The *hht1,2-K4R* almost recapitulates the meiotic phenotype of the *set1* single mutant. The *hht1,2-K4R* mutant shows reduced DSBs and is defective in SC assembly. Rad51/Dmc1 staining (Figure 7D and 7E) shows that steady state number of Rad51 foci in the *hht1,2-K4R* mutant is, on average, 26±6.8 (n  =  143) at 6 h (Figure 7F; statistically significant from numbers at 3 h in wild type, P value  =  1.4 × 10^-5^, Mann-Whitney's *U*-test), indicating the role of H3K4 in DSB formation. Moreover, the *hht1,2-K4R* mutant with the *dot1* mutation, with a greater reduction in spore viability (63.5%) compared to the wild type, shows a greater reduction in Rad51-focus number with 17±5.6 (n  =  140) at 6 h (versus wild type; P value ≈ 0, Mann-Whitney's *U*-test). Rad51 focus number in the *hht1,2-K4R dot1* mutant is 65% of the number in the *hht1,2-K4R* mutant. These support the notion that the Dot1 plays a role in DSB formation in the absence of H3K4 methylation.

The *hht1,2-K4R* double mutant is also defective in Zip1 elongation, and therefore in SC formation like the *set1* mutant (Figure 7G and 7I). The *hht1,2-K4R dot1* mutant shows more delay in SC disassembly compared to *hht1,2-K4R* (Figure 7G and 7H). We also found that the *hht1,2-K4R* double mutant often shows 2 parallel Hop1 lines like the *set1* mutant (Figure 7G). These strongly suggest a role for Set1 in SC formation as well as DSB formation through the methylation of histone H3K4.

### Histone H3K79 is critical for DSB formation in the absence of *SET1*

**Figure 8:**
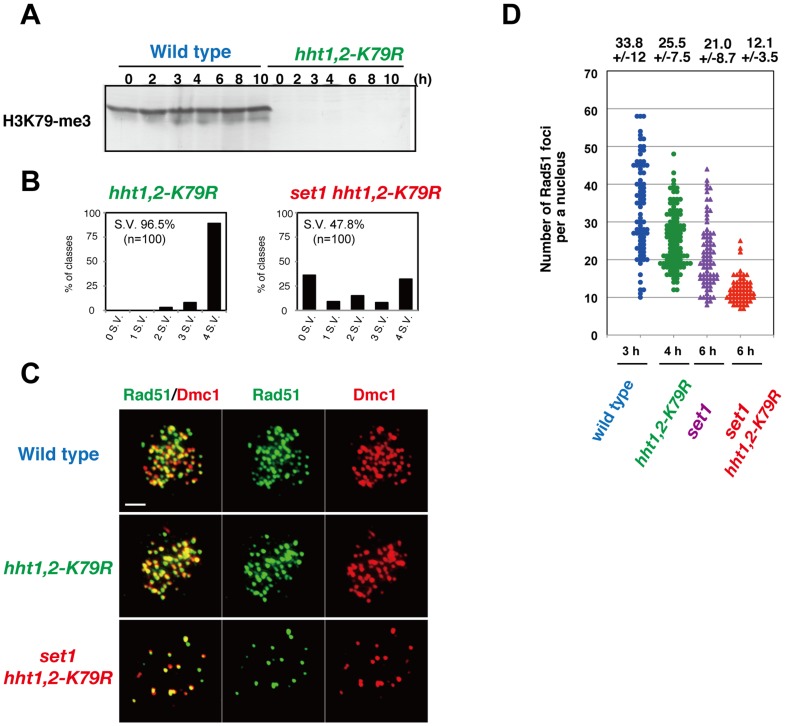
Histone H3K79 is critical for DSB without Set1. (A) Expression of histone H3K79-methylation during meiosis. Western blotting analysis for the wild-type (NKY1303/1543) and *hht1,2-K79R* (MBY151/152) strains was carried out using anti-H3K79-methylation. (B) Distribution of viable spores per tetrad in the *hht1,2-K79R* (MBY151/152) and *set1 hht1,2-K79R* (MBY219/221) strains. For each strain, 100 tetrads were dissected. (C) Immunostaining analysis of Rad51 (green) and Dmc1 (red) in wild-type cells at 3 h (NKY1303/1543) and *set1 hht1,2-K79R* (MBY219/221) cells at 6 h. The bar indicates 2 µm. (D) Rad51 focus numbers per nucleus were counted in different strains. Wild type (blue circles; NKY1303/1543), *hht1,2-K79R* (green circles; MBY219/221), the *set1* (purple triangles; MBY015/016) and *hht1,2-K79R set1* (red triangle; MBY219/221). Both the *set1* and *set1 hht1,2-K79R* mutants show delayed appearance of Rad51 foci on chromosomes. Thus, focus numbers of Rad51 at 6 h was measured. The number for the *set1* is the same as that in Figure 3C. The average number of foci with SD is shown per positive nucleus.

In order to know the involvement of histone H3K79-methylation in DSB formation, we also used a strain with histone H3K79R mutations at the native chromosomal loci (*hht1-K79R hht2-K79R*, hereafter *hht1,2-K79R*; Takehiko Usui and A.S., unpublished). The absence of H3K79 methylation was confirmed by western blot analysis using an anti-histone H3K79 methylation antibody (Figure 8A). The *hht1,2-K79R* mutant shows wild-type spore viability (Figure 8B). Importantly, when *hht1,2-K79R* was combined with the *set1* deletion, the triple mutant shows 47.8% spore viability (Figure 8B), similar to the *set1 dot1* double mutant (see Figure 1). We found that the *set1 hht1,2-K79R* mutant shows a decreased number of Rad51 foci (Figure 8C and 8D; 12.1±3.5; n  =  62), which is more reduced in the *set1* mutant. This supports the idea that Dot1-dependent histone H3K79 methylation promotes meiotic DSB formation in the absence of Set1-dependent histone H3K4 methylation. The Rad51 focus number in the *set1 hht1,2-K79R* mutant is smaller than that in the *set1 dot1* mutant (Figure 8C and 8D; versus 6 h, P value  =  1.5 × 10^−5^, Mann-Whitney's *U*-test). This may be due to culture-to culture difference.

## Discussion

Previous studies have shown that 2 histone-modifications, H3K4 methylation and H2BK123 ubiquitination, play a critical role in the formation of meiotic DSBs [7], [12], [13], [39]. The effect of H2BK123 ubiquitination seems to be indirect since this mark promotes H3K4 methylation *in trans* [38]. In this study, we have demonstrated the role of Dot1 H3K79 methyltransferase in DSB formation in the absence of Set1.

Cytological analysis of Rad51 foci, which mark sites of ongoing recombination [47], showed that, even in the absence of Set1-dependent H3K4-methylation, meiotic cells form significant numbers of DSBs, about 2/3–3/4 of the levels seen in wild type, consistent with high spore viability of the mutants defective in H3K4 methylation. In contrast, previous studies using whole genome mapping showed a large reduction of DSBs at some regions in the absence of either *SET1* or H3K4 methylation [7]. In addition, the lack of Set1-dependent H3K4 methylation changes the distribution of DSBs along chromosomes [7], [12]. However, such studies used ChIP-chip for mapping using *dmc1* or *rad50S* (*sae2*) mutants which block recombination; therefore, it is very difficult to quantify/estimate how much DSBs are dependent on the specific histone marks. Our results of Southern blotting for individual loci (Figure 2D and 4G) confirm the previous results. It is reported that the *set1* mutant showed increased DSBs at a specific chromosomal locus [7], [12], suggesting the presence of a backup system for DSB formation. We used counting of Rad51 foci to get a rough estimate of DSB numbers in a nucleus. Our ongoing research showed that the number of Rad51 foci is roughly proportional to the number of DSBs (M. S., unpublished). We found that the *set1* mutant showed a mild reduction of Rad51 focus numbers along the genome. Indeed, DSB mapping on individual chromosomes in the mutant support the idea (Figure 4). Thus, we believe that the contribution of Set1-dependent H3K4 methylation to DSB formation is weaker than expected, at least in the budding yeast. These data suggest the presence of other critical determinants for DSB formation. Indeed, we found that elimination of Dot1-dependent H3K79 methylation reduces DSB levels to about half of that seen in the *set1* deletion mutant. This indicates the involvement of the histone post-translational modification in DSB formation. In the fission yeast, H3K9 acetylation is known to promote DSB formation, while H3K4 methylation is not involved in DSB formation [9]. Moreover, in mice, meiosis-specific Prdm9-dependent H3K4 methylation shapes hotspot activity for recombination. Interestingly, even the absence of Prdm9 methyltransferase changes the distribution of the hotspot by creating new spots [11]. In Prdm9 KO mice, Prdm9-INdependent H3K4 methylation might be responsible for this activity [11]. These studies confirm that multiple histone post-translational modifications determine the site of initiation of meiotic recombination. We want to stress that, even in the absence of both Set1 and Dot1, mutant cells form 40–50% of the wild-type levels of Rad51 foci, likely DSBs, suggesting the presence of other determinant(s) for hotspot activity. Recently, it is shown that, in a plant, *Arabidopsis thaliana*, a histone H2A variant, H2A.Z, plays a role in recombination hotspot activity during meiosis [60].

Our results suggest that H3K79 methylation plays a role in DSB formation. H3K79 methylation is recognized by the Tudor domain of Rad9 in yeast [61]. Since the *rad9* mutant is proficient in meiosis [62], it is unlikely that Rad9 plays a role in DSB formation. However, we need to analyze a *rad9* mutant with the *set1* deletion to know the exact role of this protein in DSB formation, since the effect of the *dot1* is only seen in the absence of the *SET1*. Alternatively, the other protein involved in DSB formation may recognize this mark. Recent reports suggest a role for Dot1-dependent H3K79-methylation in the recombination checkpoint during meiosis [63]. In the recombination checkpoint, Dot1-dependent H3K79 methylation promotes the efficient binding of the Hop1 protein in the *zip1* mutant. This could be interpreted simply as that H3K79 methylation is bound to Hop1. However, this is unlikely, since we showed that the *dot1* mutant is proficient in Hop1 binding at least in the wild-type background. In wild type meiotic cells, there is another pathway to recruit Hop1 in a Dot1-independent manner. Recently, Dot1 has been shown to play a role in the Tel1/ATM pathway in meiotic recombination [31], [63], which somehow controls DSB formation [49], [50], [64]. If this is true in wild-type meiosis, the role of Dot1 in DSB formation described here is indirect; e.g. signaling. Indeed, recent genome-wide mapping showed that H3K79 methylation is less in promoter regions than coding regions [65]. This suggests that the Dot1-dependent H3K79 methylation play a negative role rather than a positive role in the DSB formation. We suggest that meiotic chromosomes adapt different alternatives to create the recombination hotspot, possibly using different histone marks. This kind of multiple alternatives or flexibility may contribute to the rapid evolution of the recombination hotspots.

The effect of the *dot1* mutation on DSB formation is clearly seen in the absence of H3K4 methylation. In this line, it is interesting to see subtle effects of the *dot1* mutation on DSB formation in the presence of H3K4 methylation. This includes altered kinetics of DSB repair (Figure 2D), slight but significant reduction of Rad51 focus number (Figure 3C) and the appearance of late DSBs formation (Figure 4A) in the *dot1* mutant. We need further careful evaluation on the role of the Dot1-dependent H3K79 mutation on DSB formation.

Although our studies described here suggest a direct link of Dot1 with DSB formation in the absence of Set1, we cannot exclude the possibility that the effect of *dot1* mutation is indirect through the transcription [37]. We also need more careful evaluation on the role of any histone posttranslational modifications in meiotic recombination such as DSB formation.

We also revealed a role for Set1-dependent H3K4 methylation in chromosome morphogenesis in meiosis, such as SC formation. Both the *set1* and *hht1,2-K4R* mutants produce viable spores and retain high levels of DSBs relative to wild type, as judged from the number of Rad51 foci. However, the 2 mutants are almost defective in SC elongation. The SC elongation defect in the *set1* and *hht1,2-K4R* mutants reflects persistent loading of Hop1, which often forms a linear line. The defect in SC elongation in the mutants may be caused by abnormal assembly of chromosome axes. This idea is supported by the accumulation of several axis proteins such as Rec8 and Red1 as abnormal aggregates. This abnormal assembly of the axis proteins has not been seen in other mutants defective in synapsis; e.g., *zip1* or *dmc1* mutants [59]. Thus, the recombination defect cannot account for the accumulation of this aggregate. Set1-dependent H3K4 methylation may promote the assembly of Red1 or Rec8 in the context of meiotic chromosomes.

Alternatively, reduced DSBs might be directly linked with a defect in SC elongation. In this scenario, excess DSBs in wild-type cells are necessary for normal levels of chromosome synapsis rather than recombination. This idea is somehow consistent with previous proposal of 2 types of DSBs; one for synapsis and the other for recombination [66], [67]. Indeed, a moderate reduction in DSBs does not affect the frequency of COs due to CO homeostasis [68].

## Supporting Information

Table S1 **Strain list.**
(PDF)

Table S2 **Primer list.**
(PDF)

## References

[1] Petronczki M, Siomos MF, Nasmyth K (2003) Un menage a quatre: the molecular biology of chromosome segregation in meiosis. Cell 112: 423–440. 12600308 10.1016/s0092-8674(03)00083-7

[2] Kleckner N (2006) Chiasma formation: chromatin/axis interplay and the role(s) of the synaptonemal complex. Chromosoma 115: 175–194. 16555016 10.1007/s00412-006-0055-7

[3] Borde V, de Massy B (2013) Programmed induction of DNA double strand breaks during meiosis: setting up communication between DNA and the chromosome structure. Curr Opin Genet Dev 23: 147–155. 23313097 10.1016/j.gde.2012.12.002

[4] Keeney S (2001) Mechanism and control of meiotic recombination initiation. Curr Top Dev Biol 52: 1–53. 11529427 10.1016/s0070-2153(01)52008-6

[5] Lichten M, de Massy B (2011) The impressionistic landscape of meiotic recombination. Cell 147: 267–270. 22000007 10.1016/j.cell.2011.09.038 PMC3263351

[6] Yamada T, Ohta K (2013) Initiation of meiotic recombination in chromatin structure. J Biochem. 10.1093/jb/mvt054 23750029

[7] Borde V, Robine N, Lin W, Bonfils S, Geli V, et al (2009) Histone H3 lysine 4 trimethylation marks meiotic recombination initiation sites. EMBO J 28: 99–111. 19078966 10.1038/emboj.2008.257 PMC2634730

[8] Buard J, Barthes P, Grey C, de Massy B (2009) Distinct histone modifications define initiation and repair of meiotic recombination in the mouse. EMBO J 28: 2616–2624. 19644444 10.1038/emboj.2009.207 PMC2738703

[9] Yamada S, Ohta K, Yamada T (2013) Acetylated Histone H3K9 is associated with meiotic recombination hotspots, and plays a role in recombination redundantly with other factors including the H3K4 methylase Set1 in fission yeast. Nucleic Acids Res 41: 3504–3517. 23382177 10.1093/nar/gkt049 PMC3616738

[10] Sollier J, Lin W, Soustelle C, Suhre K, Nicolas A, et al (2004) Set1 is required for meiotic S-phase onset, double-strand break formation and middle gene expression. EMBO J 23: 1957–1967. 15071505 10.1038/sj.emboj.7600204 PMC404324

[11] Brick K, Smagulova F, Khil P, Camerini-Otero RD, Petukhova GV (2012) Genetic recombination is directed away from functional genomic elements in mice. Nature 485: 642–645. 22660327 10.1038/nature11089 PMC3367396

[12] Sommermeyer V, Beneut C, Chaplais E, Serrentino ME, Borde V (2013) Spp1, a member of the Set1 Complex, promotes meiotic DSB formation in promoters by tethering histone H3K4 methylation sites to chromosome axes. Mol Cell 49: 43–54. 23246437 10.1016/j.molcel.2012.11.008

[13] Acquaviva L, Szekvolgyi L, Dichtl B, Dichtl BS, de La Roche Saint Andre C, et al (2013) The COMPASS subunit Spp1 links histone methylation to initiation of meiotic recombination. Science 339: 215–218. 23160953 10.1126/science.1225739

[14] Hunter N, Kleckner N (2001) The single-end invasion: an asymmetric intermediate at the double-strand break to double-holliday junction transition of meiotic recombination. Cell 106: 59–70. 11461702 10.1016/s0092-8674(01)00430-5

[15] Bishop DK, Park D, Xu L, Kleckner N (1992) *DMC1*: a meiosis-specific yeast homolog of *E. coli recA* required for recombination, synaptonemal complex formation, and cell cycle progression. Cell 69: 439–456. 1581960 10.1016/0092-8674(92)90446-j

[16] Cloud V, Chan YL, Grubb J, Budke B, Bishop DK (2012) Rad51 is an accessory factor for Dmc1-mediated joint molecule formation during meiosis. Science 337: 1222–1225. 22955832 10.1126/science.1219379 PMC4056682

[17] Shinohara A, Ogawa H, Ogawa T (1992) Rad51 protein involved in repair and recombination in *S. cerevisiae* is a RecA-like protein. Cell 69: 457–470. 1581961 10.1016/0092-8674(92)90447-k

[18] Schwacha A, Kleckner N (1994) Identification of joint molecules that form frequently between homologs but rarely between sister chromatids during yeast meiosis. Cell 76: 51–63. 8287479 10.1016/0092-8674(94)90172-4

[19] Allers T, Lichten M (2001) Differential timing and control of noncrossover and crossover recombination during meiosis. Cell 106: 47–57. 11461701 10.1016/s0092-8674(01)00416-0

[20] Zickler D, Kleckner N (1999) Meiotic chromosomes: integrating structure and function. Annu Rev Genet 33: 603–754. 10690419 10.1146/annurev.genet.33.1.603

[21] Borner GV, Kleckner N, Hunter N (2004) Crossover/noncrossover differentiation, synaptonemal complex formation, and regulatory surveillance at the leptotene/zygotene transition of meiosis. Cell 117: 29–45. 15066280 10.1016/s0092-8674(04)00292-2

[22] Hochwagen A, Amon A (2006) Checking your breaks: surveillance mechanisms of meiotic recombination. Curr Biol 16: R217–228. 16546077 10.1016/j.cub.2006.03.009

[23] Sym M, Engebrecht JA, Roeder GS (1993) ZIP1 is a synaptonemal complex protein required for meiotic chromosome synapsis. Cell 72: 365–378. 7916652 10.1016/0092-8674(93)90114-6

[24] Xu L, Ajimura M, Padmore R, Klein C, Kleckner N (1995) *NDT80*, a meiosis-specific gene required for exit from pachytene in *Saccharomyces cerevisiae* . Mol Cell Biol 15: 6572–6581. 8524222 10.1128/mcb.15.12.6572 PMC230910

[25] Chu S, Herskowitz I (1998) Gametogenesis in yeast is regulated by a transcriptional cascade dependent on Ndt80. Mol Cell 1: 685–696. 9660952 10.1016/s1097-2765(00)80068-4

[26] Clyne RK, Katis VL, Jessop L, Benjamin KR, Herskowitz I, et al (2003) Polo-like kinase Cdc5 promotes chiasmata formation and cosegregation of sister centromeres at meiosis I. Nat Cell Biol 5: 480–485. 12717442 10.1038/ncb977

[27] Sourirajan A, Lichten M (2008) Polo-like kinase Cdc5 drives exit from pachytene during budding yeast meiosis. Genes Dev 22: 2627–2632. 18832066 10.1101/gad.1711408 PMC2559907

[28] San-Segundo PA, Roeder GS (1999) Pch2 links chromatin silencing to meiotic checkpoint control. Cell 97: 313–324. 10319812 10.1016/s0092-8674(00)80741-2

[29] San-Segundo PA, Roeder GS (2000) Role for the silencing protein Dot1 in meiotic checkpoint control. Mol Biol Cell 11: 3601–3615. 11029058 10.1091/mbc.11.10.3601 PMC15018

[30] Borner GV, Barot A, Kleckner N (2008) Yeast Pch2 promotes domainal axis organization, timely recombination progression, and arrest of defective recombinosomes during meiosis. Proc Natl Acad Sci U S A 105: 3327–3332. 18305165 10.1073/pnas.0711864105 PMC2265181

[31] Ho HC, Burgess SM (2011) Pch2 acts through Xrs2 and Tel1/ATM to modulate interhomolog bias and checkpoint function during meiosis. PLoS Genet 7: e1002351. 22072981 10.1371/journal.pgen.1002351 PMC3207854

[32] Conde F, Ontoso D, Acosta I, Gallego-Sanchez A, Bueno A, et al (2010) Regulation of tolerance to DNA alkylating damage by Dot1 and Rad53 in *Saccharomyces cerevisiae* . DNA Repair (Amst) 9: 1038–1049. 20674515 10.1016/j.dnarep.2010.07.003

[33] Conde F, Refolio E, Cordon-Preciado V, Cortes-Ledesma F, Aragon L, et al (2009) The Dot1 histone methyltransferase and the Rad9 checkpoint adaptor contribute to cohesin-dependent double-strand break repair by sister chromatid recombination in *Saccharomyces cerevisiae* . Genetics 182: 437–446. 19332880 10.1534/genetics.109.101899 PMC2691753

[34] Conde F, San-Segundo PA (2008) Role of Dot1 in the response to alkylating DNA damage in *Saccharomyces cerevisiae*: regulation of DNA damage tolerance by the error-prone polymerases Polzeta/Rev1. Genetics 179: 1197–1210. 18562671 10.1534/genetics.108.089003 PMC2475726

[35] Levesque N, Leung GP, Fok AK, Schmidt TI, Kobor MS (2010) Loss of H3 K79 trimethylation leads to suppression of Rtt107-dependent DNA damage sensitivity through the translesion synthesis pathway. J Biol Chem 285: 35113–35122. 20810656 10.1074/jbc.M110.116855 PMC2966125

[36] Tatum D, Li S (2011) Evidence that the histone methyltransferase Dot1 mediates global genomic repair by methylating histone H3 on lysine 79. J Biol Chem 286: 17530–17535. 21460225 10.1074/jbc.M111.241570 PMC3093827

[37] Nguyen AT, Zhang Y (2011) The diverse functions of Dot1 and H3K79 methylation. Genes Dev 25: 1345–1358. 21724828 10.1101/gad.2057811 PMC3134078

[38] Shilatifard A (2012) The COMPASS family of histone H3K4 methylases: mechanisms of regulation in development and disease pathogenesis. Annu Rev Biochem 81: 65–95. 22663077 10.1146/annurev-biochem-051710-134100 PMC4010150

[39] Yamashita K, Shinohara M, Shinohara A (2004) Rad6-Bre1-mediated histone H2B ubiquitylation modulates the formation of double-strand breaks during meiosis. Proc Natl Acad Sci U S A 101: 11380–11385. 15280549 10.1073/pnas.0400078101 PMC509210

[40] Shinohara M, Gasior SL, Bishop DK, Shinohara A (2000) Tid1/Rdh54 promotes colocalization of Rad51 and Dmc1 during meiotic recombination. Proc Natl Acad Sci U S A 97: 10814–10819. 11005857 10.1073/pnas.97.20.10814 PMC27106

[41] Farmer S, Leung WK, Tsubouchi H (2011) Characterization of meiotic recombination initiation sites using pulsed-field gel electrophoresis. Methods Mol Biol 745: 33–45. 21660687 10.1007/978-1-61779-129-1_3

[42] Shinohara M, Sakai K, Ogawa T, Shinohara A (2003) The mitotic DNA damage checkpoint proteins Rad17 and Rad24 are required for repair of double-strand breaks during meiosis in yeast. Genetics 164: 855–865. 12871899 10.1093/genetics/164.3.855 PMC1462628

[43] Storlazzi A, Xu L, Cao L, Kleckner N (1995) Crossover and noncrossover recombination during meiosis: timing and pathway relationships. Proc Natl Acad Sci U S A 92: 8512–8516. 7667321 10.1073/pnas.92.18.8512 PMC41187

[44] Cao L, Alani E, Kleckner N (1990) A pathway for generation and processing of double-strand breaks during meiotic recombination in *S. cerevisiae* . Cell 61: 1089–1101. 2190690 10.1016/0092-8674(90)90072-m

[45] Schwacha A, Kleckner N (1997) Interhomolog bias during meiotic recombination: meiotic functions promote a highly differentiated interhomolog-only pathway. Cell 90: 1123–1135. 9323140 10.1016/s0092-8674(00)80378-5

[46] Shinohara A, Gasior S, Ogawa T, Kleckner N, Bishop DK (1997) *Saccharomyces cerevisiae recA* homologues *RAD51* and *DMC1* have both distinct and overlapping roles in meiotic recombination. Genes Cells 2: 615–629. 9427283 10.1046/j.1365-2443.1997.1480347.x

[47] Bishop DK (1994) RecA homologs Dmc1 and Rad51 interact to form multiple nuclear complexes prior to meiotic chromosome synapsis. Cell 79: 1081–1092. 7528104 10.1016/0092-8674(94)90038-8

[48] Miyazaki T, Bressan DA, Shinohara M, Haber JE, Shinohara A (2004) In vivo assembly and disassembly of Rad51 and Rad52 complexes during double-strand break repair. Embo J 23: 939–949. 14765116 10.1038/sj.emboj.7600091 PMC380999

[49] Carballo JA, Panizza S, Serrentino ME, Johnson AL, Geymonat M, et al (2013) Budding Yeast ATM/ATR Control Meiotic Double-Strand Break (DSB) Levels by Down-Regulating Rec114, an Essential Component of the DSB-machinery. PLoS Genet 9: e1003545. 23825959 10.1371/journal.pgen.1003545 PMC3694840

[50] Argunhan B, Farmer S, Leung WK, Terentyev Y, Humphryes N, et al (2013) Direct and indirect control of the initiation of meiotic recombination by DNA damage checkpoint mechanisms in budding yeast. PLoS One 8: e65875. 23762445 10.1371/journal.pone.0065875 PMC3677890

[51] Trelles-Sticken E, Bonfils S, Sollier J, Geli V, Scherthan H, et al (2005) Set1- and Clb5-deficiencies disclose the differential regulation of centromere and telomere dynamics in *Saccharomyces cerevisiae* meiosis. J Cell Sci 118: 4985–4994. 16254243 10.1242/jcs.02612

[52] Hollingsworth NM, Goetsch L, Byers B (1990) The *HOP1* gene encodes a meiosis-specific component of yeast chromosomes. Cell 61: 73–84. 2107981 10.1016/0092-8674(90)90216-2

[53] Wojtasz L, Daniel K, Roig I, Bolcun-Filas E, Xu H, et al (2009) Mouse HORMAD1 and HORMAD2, two conserved meiotic chromosomal proteins, are depleted from synapsed chromosome axes with the help of TRIP13 AAA-ATPase. PLoS Genet 5: e1000702. 19851446 10.1371/journal.pgen.1000702 PMC2758600

[54] Armstrong SJ, Caryl AP, Jones GH, Franklin FC (2002) Asy1, a protein required for meiotic chromosome synapsis, localizes to axis-associated chromatin in Arabidopsis and Brassica. J Cell Sci 115: 3645–3655. 12186950 10.1242/jcs.00048

[55] Rockmill B, Roeder GS (1988) *RED1*: a yeast gene required for the segregation of chromosomes during the reductional division of meiosis. Proc Natl Acad Sci U S A 85: 6057–6061. 3413075 10.1073/pnas.85.16.6057 PMC281904

[56] Klein F, Mahr P, Galova M, Buonomo SB, Michaelis C, et al (1999) A central role for cohesins in sister chromatid cohesion, formation of axial elements, and recombination during yeast meiosis. Cell 98: 91–103. 10412984 10.1016/S0092-8674(00)80609-1

[57] Hollingsworth NM, Ponte L (1997) Genetic interactions between *HOP1*, *RED1* and *MEK1* suggest that *MEK1* regulates assembly of axial element components during meiosis in the yeast *Saccharomyces cerevisiae* . Genetics 147: 33–42. 9286666 10.1093/genetics/147.1.33 PMC1208117

[58] Leem SH, Ogawa H (1992) The *MRE4* gene encodes a novel protein kinase homologue required for meiotic recombination in *Saccharomyces cerevisiae* . Nucleic Acids Res 20: 449–457. 1741279 10.1093/nar/20.3.449 PMC310407

[59] Smith AV, Roeder GS (1997) The yeast Red1 protein localizes to the cores of meiotic chromosomes. J Cell Biol 136: 957–967. 9060462 10.1083/jcb.136.5.957 PMC2132480

[60] Choi K, Zhao X, Kelly KA, Venn O, Higgins JD, et al (2013) *Arabidopsis* meiotic crossover hot spots overlap with H2A.Z nucleosomes at gene promoters. Nat Genet 45: 1327–1336. 24056716 10.1038/ng.2766 PMC3812125

[61] Grenon M, Costelloe T, Jimeno S, O'Shaughnessy A, Fitzgerald J, et al (2007) Docking onto chromatin via the *Saccharomyces cerevisiae* Rad9 Tudor domain. Yeast 24: 105–119. 17243194 10.1002/yea.1441

[62] Lydall D, Nikolsky Y, Bishop DK, Weinert T (1996) A meiotic recombination checkpoint controlled by mitotic checkpoint genes. Nature 383: 840–843. 8893012 10.1038/383840a0

[63] Ontoso D, Acosta I, van Leeuwen F, Freire R, San-Segundo PA (2013) Dot1-dependent histone H3K79 methylation promotes activation of the Mek1 meiotic checkpoint effector kinase by regulating the Hop1 adaptor. PLoS Genet 9: e1003262. 23382701 10.1371/journal.pgen.1003262 PMC3561090

[64] Gray S, Allison RM, Garcia V, Goldman AS, Neale MJ (2013) Positive regulation of meiotic DNA double-strand break formation by activation of the DNA damage checkpoint kinase Mec1(ATR). Open Biol 3: 130019. 23902647 10.1098/rsob.130019 PMC3728922

[65] Zhang L, Ma H, Pugh BF (2011) Stable and dynamic nucleosome states during a meiotic developmental process. Genome Res 21: 875–884. 21515815 10.1101/gr.117465.110 PMC3106320

[66] Stahl FW, Foss HM, Young LS, Borts RH, Abdullah MF, et al (2004) Does crossover interference count in *Saccharomyces cerevisiae* ? Genetics 168: 35–48. 15454525 10.1534/genetics.104.027789 PMC1448104

[67] Zalevsky J, MacQueen AJ, Duffy JB, Kemphues KJ, Villeneuve AM (1999) Crossing over during *Caenorhabditis elegans* meiosis requires a conserved MutS-based pathway that is partially dispensable in budding yeast. Genetics 153: 1271–1283. 10545458 10.1093/genetics/153.3.1271 PMC1460811

[68] Martini E, Diaz RL, Hunter N, Keeney S (2006) Crossover homeostasis in yeast meiosis. Cell 126: 285–295. 16873061 10.1016/j.cell.2006.05.044 PMC1949389

