# Supplementary material for: Dot1-Dependent Histone H3K79 Methylation Promotes the Formation of Meiotic Double-Strand Breaks in the Absence of Histone H3K4 Methylation in Budding Yeast
Source: PLoS One. 2014 May 5;9(5):e96648. doi: 10.1371/journal.pone.0096648 (PMC4010517; doi:10.1371/journal.pone.0096648)
Supplement: Table S1 — Strain list. (PDF) [file pone.0096648.s001.pdf]

Supplemental Table S1. Strain list

Strain No.	Genotypes
NKY1543	*MATα, ho::LYS2, lys2, ura3, leu2::hisG, his4X-LEU2(BamHI)-URA3, arg4-nsp*
NKY1303	*MAT**a**, ho::LYS2, lys2, ura3, leu2::hisG, his4B-LEU2(MluI), arg4-bgl*
MSY2632	NKY1303 with *dmc1::URA3*
MSY2630	NKY1543 with *dmc1::URA3*
MBY005	NKY1303 with *dot1::KanMX6*
MBY006	NKY1543 with *dot1::KanMX6*
MBY015	NKY1303 with *set1:: KlURA3*
MBY016	NKY1543 with *set1::KlURA3*
MBY037	NKY1303 with *dot1::KanMX6, set1:: KlURA3*
MBY039	NKY1543 with *dot1::KanMX6, set1::KlURA3*
MBY211	NKY1303 with *hht1-K4R, hht2-K4R*
MBY218	NKY1543 with *hht1-K4R, hht2-K4R*
MBY233	NKY1303 with *hht1-K4R, hht2-K4R, dot1::KanMX6*
MBY237	NKY1543 with *hht1-K4R, hht2-K4R, dot1::KanMX6*
MBY003	NKY1303 with *dmc1::URA3, dot1::KanMX6*
MBY004	NKY1543 with *dmc1::URA3, dot1::KanMX6*
MBY021	NKY1303 with *dmc1::URA3, set1::KlURA3*
MBY022	NKY1543 with *dmc1::URA3, set1::KlURA3*
MBY282	NKY1303 with *dmc1::URA3, set1::KlURA3, dot1::KanMX6*
MBY285	NKY1543 with *dmc1::URA3, set1::KlURA3, dot1::KanMX6*
MBY151	NKY1543 with *hht1-K79R, hht2-K79R*
MBY152	NKY1303 with *hht1-K79R, hht2-K79R*
MBY219	NKY1303 with *set1::KlURA3, hht1-K79R, hht2-K79R*
MBY221	NKY1543 with *set1::KlURA3, hht1-K79R, hht2-K79R*
