# Supplementary material for: Dot1-Dependent Histone H3K79 Methylation Promotes the Formation of Meiotic Double-Strand Breaks in the Absence of Histone H3K4 Methylation in Budding Yeast
Source: PLoS One. 2014 May 5;9(5):e96648. doi: 10.1371/journal.pone.0096648 (PMC4010517; doi:10.1371/journal.pone.0096648)
Supplement: Table S2 — Primer list. (PDF) [file pone.0096648.s002.pdf]

Supplemental Table S2. Primer list

Cloning for *HTT1*

5’ AGCTATCCGGAATTCGGGGGAGAAGCGCTCGGAACA 3’

5’ TCGACTCCCAAGCTTGACACCTACCACGTATGCGG 3’

K4R mutagenesis for *HHT1*

5’ ATGGCCAGAACGCGTCAAACAGCAAGA 3’

5’ TCTTGCTGTTTGACGCGTTCTCGGCAT

Cloning for *HTT2*

5’ CCGAATTCCAAACACGTATGTATCTAGCCG

5’ CCCGCGGCCGCGTGTTGAATCCTGCGAATC 3

K4R mutagenesis for *HHT2*

5’ ATGGCCAGAACGCGTCAAACAGCAAGA

5’ ATGGCCAGAACGCGTCAAACAGCAAGA

Blue letters show mutated nucleotides.
